# Coronavirus disease 2019 (COVID-19) related cytopenia: A protocol for systematic review and meta-analysis

**DOI:** 10.1097/MD.0000000000022033

**Published:** 2020-09-04

**Authors:** Yiwei Li, Qianping Weng, Xilian Huang, Yaping Xie, Can Chen, Shenxian Qian

**Affiliations:** a Department of Hematology, Affiliated Hangzhou First People's Hospital, Nanjing Medical University; b Department of Hematology, Affiliated Hangzhou First People's Hospital, Zhejiang University School of Medicine, Hangzhou, Zhejiang, PR. China.

**Keywords:** COVID-19, cytopenia, meta-analysis, protocol, systematic review

## Abstract

**Background:**

In December 2019, the novel coronavirus pneumonia was detected in Wuhan and named COVID-19. It is an international outbreak of the respiratory illness caused by severe acute respiratory syndrome coronavirus 2. Recent papers pointed out the cytopenia in COVID-19 patients including lymphopenia, neutrophilia, thrombocytopenia and lower level of hemoglobin had prognostic significance. This systemic review and meta-analysis summaries the latest evidence from available data and determine the hematological abnormality caused by severe acute respiratory syndrome coronavirus 2 and potential efficacy on the outcomes in patients with COVID-19.

**Methods:**

This protocol for a systematic reviews and meta-analysis will be performed according to the preferred reporting items for systematic reviews and meta-analysis protocols 2015 guidelines. The database of Cochrane Library, PUBMED, EMBASE, Medline, Web of Science, Google Scholar, CNKI, WanFang, as well as gray literatures from the inception to present will be comprehensively and systematically searched without limitations of regions or language. The main study outcomes will be the mortality of COVID-19 patients. The meta-analysis was performed by RevMan V.5.3 program and Stata V.12.0 software after 2 reviewers independently selected literature, data extraction, bias risk evaluation and study quality assessment. Any disagreement will be resolved by consensus to the third researcher.

**Results:**

This systematic review and meta-analysis may help provide clarify on the effect of cytopenia in patients with COVID-19. The result will be published at a peer-reviewed journal.

**Conclusions:**

This proposed study will evaluate the existing evidence on the effectiveness of cytopenia in COVID-19 patients.

**Ethic and dissemination:**

The content of this article does not involve moral approval or ethical review because no individual data will be collected.

**PROSPERO registration:**

CRD42020187524.

## 1 Introduction

Coronavirus Disease 2019 (COVID-2019), a rapid and global spread virus, has attacked people in almost all countries and posed a great threat to public health worldwide.^[1,2]^ It is a human infectious disease caused by the novel Severe Acute Respiratory Syndrome coronavirus 2 (SARS-CoV-2), which is transmitted mainly via droplets and contaminated surfaces.^[3]^ To date, there have been 16682030 confirmed cases of COVID-19, including 659374 deaths, reported to World Health Organization.^[4]^

SARS-CoV-2 is a class of enveloped, positive single-stranded RNA virus, which has shown similar genome sequence, receptor affinity, pathogenesis and disease presentation with its predecessors including SARS-CoV and MERS-CoV. However, our knowledge about MERS or SARA may not be enough to manage current COVID-19 pandemic. Patients most frequently present with infectious may be with no or mild pneumonia. A small proportion of the patients progressed to severe lung injury, respiratory failure, acute respiratory distress syndrome and multiple organ failure requiring hospitalization in intensive care unit that associated with a high mortality rate.^[5]^ Thus, early identification of individuals at high risk of developing severe symptoms may offer a better clinical management so at to improve outcomes.

It is well known that immune dysregulation and cytokine storms play the significant role in the severity assessment of the patients with SARS and MERS.^[6]^ Several factors have been proposed trying to predict the severity of the disease. Recent available publications are accumulating evidence suggesting that blood count abnormalities commonly occurred in COVID-19, including lymphopenia, neutrophilia, thrombocytopenia, and lower level of hemoglobin.^[7–11]^ Moreover, some retrospective analysis showed hematological differences exist with respect to the severity of COVID-19.^[10–12]^ In addition, higher neutrophil count and neutrophil-to-lymphocyte ratio, and lower hemoglobin concentration have shown to be risk factors for severe illness in patients with SARS-CoV-2 infection.^[13]^ Since considerable amount of retrospective studies have primarily been published, we conducted the present meta-analysis to investigate the prognostic effect of cytopenia on patients with COVID-19.

## 2 Materials and methods

### 2.1 Study registration

This study will be conducted in accordance with the preferred reporting items for systematic reviews and meta-analysis protocols 2015 guidelines and the protocol has been registering in the PROSPERO database (Registration number: CRD42020187524) on 22 May 2020.^[14]^

### 2.2 Inclusion criteria

#### 2.2.1 Types of studies

The present study will include observational, retrospective and prospective studies, cross-sectional studies or clinical trials without language, date, or publication status restrictions.

#### 2.2.2 Types of participants

Adult patients who presented COVID-19 pneumonia associated with cytopenia. There are no restrictions on the region, gender and disease severity.

#### 2.2.3 Outcome measures

The primary outcome will be the mortality of COVID-19 patients. Secondary outcomes will include the baseline and nadir of blood cell counts, proportion of patients requiring mechanical ventilation or hemodynamic support, proportion of patients with adverse events (sepsis, life-threatening bleeding, multiple organ dysfunction syndrome, etc), proportion of patients admitted to ICU, length of stay in hospital.

### 2.3 Exclusion criteria

The exclusion criteria are as follows:

(1) Patients with history of disease that may lead to cytopenia including (but not limit): idiopathic thrombocytopenic purpura, myelodysplastic syndrome, hematological malignancy, immunologic thrombocytopenia, any type of anemia or any drug induced cytopenia identified.
(2) Studies with overlapping data.
(3) Conference reports, thesis, reviews, case reports, letters to the editor, editorials and expert opinions.
(4) Studies that enrolled less than 10 patients.
(5) Missing or insufficient data that cannot be obtained after contacting original authors.

### 2.4 Search strategy

We will search following electronic databases for relevant studies: Cochrane Library, PUBMED, EMBASE, MEDLINE, SCI-EXPANDED, Web of Science, Google Scholar, CNKI, WanFang database up to July 15, 2020. In addition, gray literatures will also be searched, including conference proceedings and reference lists of included studies. The searching strategy for PUBMED have been shown in Table 1.

**Table 1:**
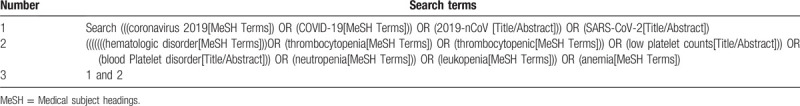
Search strategy used in Pubmed database.

#### 2.4.1 Other resources

**Figure 1:**
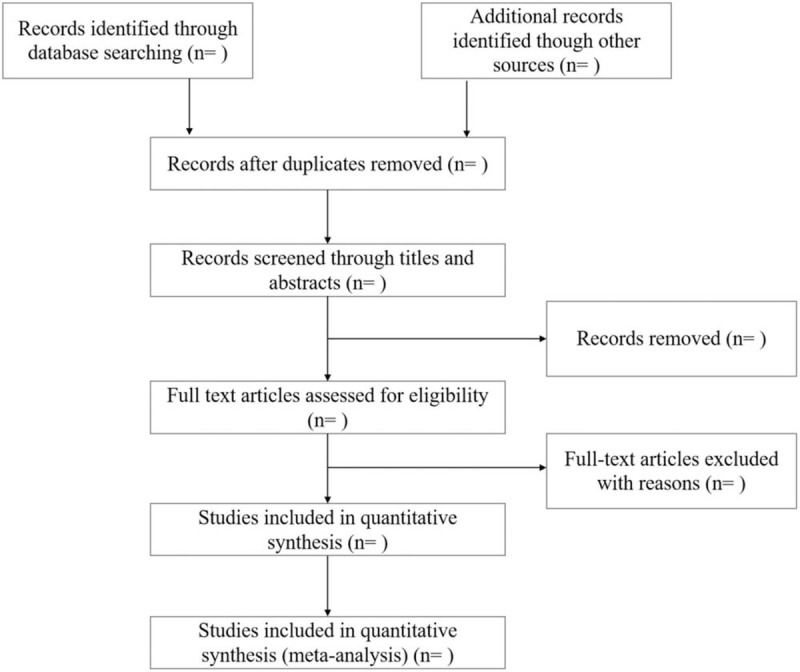
Flow diagram of study selection process.

Similar retrieval methods will be applied in the COVID-19 Study Registry (https://covid-19.cochrane.org/) and COVID-evidence (https://covid-evidence.org/) to obtain unpublished studies. There is no restriction on publication regions or language. The process of study selection is illustrated following a preferred reporting items for systematic reviews and meta-analysis guidelines (Fig. 1).

### 2.5 Study selection

Reference lists will be collected in Endnote X9 software and duplicate articles will be deleted. Then 2 reviewers will independently scan the titles and abstracts of the retrieved articles to exclude obvious irrelevant studies. The full text of potentially relevant studies will then be reviewed in accordance with the prespecified criteria by 2 reviewers. If authors are similar or data are extracted from the same database, the study period will be noted. Only the latest study will be included if the study period overlaps. Any disagreement between authors will be resolved by consensus with a third author.

### 2.6 Data extraction and management

The data will be collected by 2 independent reviewers from all eligible studies using a standardized data extraction sheet. It consists of following information: title, author details, publication time, country, study design and setting, publication status, participant characteristics, eligible criteria, outcomes, risk of bias and other essential information. To ensure the accuracy and consistency of the extracted data, cross-checked will be used by 2 reviewers. Any dispute in the data extraction process will be solved by a third reviewer.

### 2.7 Dealing with missing data

For any data missing or clarifications needed, we will contact original trial authors to request them. Otherwise, we will analyze the available data if we cannot achieve them.

### 2.8 Risk of bias assessment

The risk of bias in enrolled studies will be independently assessed by 2 reviewers, any discrepancy will be resolved by consulting a third reviewer. Newcastle-Ottawa Scale will be used to evaluate risk of bias for each case-control studies and cohort studies, which consisted of 3 parameters: selection, comparability, and exposure assessment. The potential risk of bias in each clinical trial will be evaluated by Cochrane collaboration tool through 7 domains. Each of the domains will be scored as “low risk”, “high risk” or “Unclear”.^[15]^

### 2.9 Data synthesis

We will use RevMan V.5.3 program and Stata V.12.0 software to process statistical analysis. Dichotomous outcomes will be determined by using risk ratio with confidence intervals of 95%. For continuous variables will be recorded as the mean differences with 95% confidence intervals. To determine the impact of the statistical heterogeneity on the meta-analysis, we will primarily use forest plots to assess any sign of potential heterogeneity visually. All statistical heterogeneity will be appraised by Cochran *Q* and Higgins *I*^2^ test. A *P* value of <.10 for the Chi^2^statistic or an *I*^2^ > 50% indicates significant heterogeneity, Further analysis will be carried out to investigate the possible sources of heterogeneity. After excluding the influence of obvious heterogeneity, the random-effect model will be placed. If necessary, we will report study results by narrative description. A *P* value of >.10 or an *I*^2^ < 50% indicates no statistical heterogeneity among the studies, and the fixed-effect model will be applied, and we will consider conducting meta-analysis.

### 2.10 Investigation of heterogeneity

#### 2.10.1 Meta-regression or subgroup analysis

If the number of studies is sufficient, subgroup analysis or meta-regression will be designed to explore causes of heterogeneity observed in the primary analysis, including population characteristics, study design and research quality, sample size, demographic characters, follow-up period.

#### 2.10.2 Sensitivity analysis

To ensure the stability of the results, we will undertake sensitivity analysis of the results by excluding each of the studies included in the analysis 1 by 1 and repeat the analysis and comparing the differences between the re-obtained results and the original results. In this way, we will be able to examine the impact of individual studies in the overall effect and their robustness.

### 2.11 Reporting bias analysis

The integrity of the eligible studies is mainly assessed by reporting bias, of which publication bias is the most common. We will generate funnel plot and Egger regression test to detect reporting bias after confirming the eligible studies are adequate (≥10). Funnel plot will be asymmetry, or a *P* value of Egger regression test will be less than 0.05 when publication bias exists.^[16]^

### 2.12 Quality assessment of the cumulative evidence

The overall quality of evidence will be assessed by the Grading of recommendations, assessment, development, and evaluation system. The quality of each included study will be independently evaluated by 2 reviewers. Any discrepancy will be adjudicated by a third reviewer. The evidence quality will be graded as high, moderate, low or very low according to 5 parameters (publication bias, indirectness, inconsistency, imprecision, and study limitations).^[17]^

## 3 Discussion

Emerging studies have reported that hematological abnormality in patients who experience with COVID-19 was commonly and several markers may be predictive of disease severity. The underlying mechanism is likely to be some biomarkers may be positively correlated with increased proinflammatory cytokines and immune dysregulation. However, not all studies of cytopenia in COVID-19 have reported completely consistent findings, nor have comprehensively measure related evidence. Thus, we conduct the present meta-analysis to investigate the prognostic effect of cytopenia on patients with COVID-19. This protocol will be divided into 5 sections: identification, study inclusion, data extraction, data synthesis and study quality assessment. We believe that this meta-analysis will provide information on the association between cytopenia and clinical outcome of COVID-19 patients.

## Author contributions

**Conceptualization:** Shenxian Qian, Can Chen

**Data curation:** Yiwei Li, Qianping Weng.

**Formal analysis:** Qianping Weng, Xilian Huang.

**Funding acquisition:** Shenxian Qian.

**Investigation:** Yiwei Li, Yaping Xie.

**Methodology:** Xilian Huang, Yaping Xie.

**Software:** Yiwei Li, Qianping Weng.

**Supervision:** Shenxian Qian.

**Writing – original draft:** Yiwei Li, Qianping Weng, Xilian Huang, Yaping Xie, Can Chen

**Writing – review & editing:** Yiwei Li, Qianping Weng, Xilian Huang, Yaping Xie, Can Chen, Shenxian Qian.
